# A rare manifestation of adrenocortical carcinoma as a mimic of pheochromocytoma: a case report and literature review

**DOI:** 10.3389/fendo.2025.1533711

**Published:** 2025-02-11

**Authors:** Joanna Sobolewska, Wioleta Respondek, Przemyslaw Witek

**Affiliations:** ^1^ Department of Internal Medicine, Endocrinology and Diabetes, Medical University of Warsaw, Warsaw, Poland; ^2^ Department of Internal Medicine, Endocrinology and Diabetes, Mazovian Brodnowski Hospital, Warsaw, Poland

**Keywords:** adrenocortical carcinoma, pheochromocytoma, adrenal incidentaloma, symptoms, mild autonomous cortisol secretion

## Abstract

The primary management in the care of patients with adrenal incidentalomas is to determine the oncologic risk, namely, the possibility of malignancy. The first place among adrenal incidentaloma lesions requiring diagnosis and treatment promptly is adrenocortical carcinoma (ACC). Similarly, in the case of pheochromocytoma, the lack of early diagnosis worsens the patient’s prognosis. Even though both ACC and pheochromocytoma are among the less frequent adrenal lesions, neither should be excluded during differential diagnostics, especially in patients with an equivocal clinical presentation and non-typical adenoma radiological features. ACC presenting as pheochromocytoma is one of the few cases described in the literature, some of which could not collect exhaustive clinical data. Herein, in this article, we would like to provide an overview of reported ACC cases clinically manifesting as pheochromocytoma, based on the clinical image of a 59-year-old female patient with unintentional weight loss, non-specific abdominal pain, a diagnosis of hypertension, and significantly elevated excretion of 3-methoxytyramine in a 24-h urine collection, histopathologically diagnosed with ACC. The case presented emphasizes how crucial a comprehensive diagnostics and individual approach to the patient would be.

## Introduction

Adrenal incidentalomas are adrenal masses detected on imaging studies performed for reasons other than suspected adrenal disease. The etiology of adrenal focal lesions is heterogeneous and includes both benign and malignant lesions originating from the adrenal cortex, medulla, and masses of extra-adrenal source (1). Autopsy evaluation indicates the prevalence of adrenal lesions at approximately 2%, increasing with age (1). The most relevant diagnostic tools for adrenal tumors are unenhanced computed tomography (CT), magnetic resonance imaging (MRI) with technique of chemical-shift imaging, and ^18^F-fluorodeoxyglucose positron emission tomography/CT (^18^F-FDG-PET-CT) (2). Abdominal ultrasound remains of limited importance (1, 2). In adrenal imaging, MRI is the preferred choice over CT in children, adolescents, and pregnant women (1). A special position in adrenal incidentaloma management is given to lesions of potential malignancy, which, on unenhanced CT, are manifested by the presence of heterogeneous adrenal lesions with a diameter ≥4cm and a density exceeding 10 Hounsfield units (HU), usually >20 HU (1). Two primary malignant lesions may originate from the adrenal glands—adrenocortical carcinoma (ACC) and pheochromocytoma, both of which remain infrequent lesions (1, 3). ACC is a malignant tumor with an unfavorable prognosis (2), which is estimated to occur in 0.5–2 people per million per year (3). The ACC accounts for 0.4% to 4% of all adrenal incidentalomas (1), and its peak incidence occurs in the fifth and sixth decades of life (4). In 30%–40% of ACC cases, the diagnosis is led by clinical manifestations due to an excess of cortisol, less frequently androgens, and most rarely mineralocorticosteroids (2). The characteristic features of ACC in the CT, besides density exceeding 10 HU in the native phase, comprise necrotic and hemorrhagic areas visible in the tumor (2).The majority of ACC are sporadic, but 5%–10% may arise in the course of germline mutations, such as Li–Fraumeni syndrome, Lynch syndrome, and several cancer hereditary predisposition syndromes—multiple endocrine neoplasia type 1, neurofibromatosis type 1, familial adenomatous polyposis, Gardner syndrome, Beckwith–Wiedemann syndrome, and Carney complex (2, 5). Because of this, molecular diagnostics’ role in managing patients with ACC seems particularly encouraging. The investigation should include comorbid cancers and medical family history, while clinicians must individualize screening based on the gene of interest and age of onset of related cancers (2, 6). Any suspected cancer hereditary predisposition syndromes should involve referring the patient for genetic testing, which, if Lynch syndrome is confirmed, may open up other therapeutic options for the patient, such as immunotherapy with immune checkpoint inhibitors (2, 6).

Pheochromocytoma is a neuroendocrine tumor producing catecholamines, originating in the chromaffin cells of the adrenal medulla or extra-adrenal paraganglia (3, 7). Pheochromocytoma accounts for 1%–5% of all adrenal incidentalomas (1). Pheochromocytoma and paraganglioma are described collectively as PPGL group, with an incidence of two to eight cases per million people per year (3). Pheochromocytoma should always be considered in the differential diagnosis because of the risk of causing a life-threatening catecholamine crisis and specific management before surgical treatment (3). The risk of metastatic lesions of PPGL increases with a tumor ≥50 mm in diameter, the presence of paraganglioma with extra-adrenal localization, a germline mutation of the iron sulfur B subunit of the succinate dehydrogenase complex (SDHB), and plasma 3-methoxytramine (3MT) levels more than three times the upper reference limit (URL) (3). The gene that encodes the B subunit of the SDHB complex remains a key molecular contributor to malignant PPGL, with mutations in this gene found in at least 40% of all metastatic PPGL cases (3). Vigilance should be maintained, especially when the clinical picture is ambiguous. Therefore, we would like to present the case of a patient with ACC, clinically manifested as pheochromocytoma, and a review of similar cases available in the literature. The indicated case is one of the few described, so we would like to emphasize the role of interdisciplinary cooperation, extensive diagnostics, and individual approach in the care of patients with adrenal lesions.

## Case presentation

A 59-year-old female patient with incidentally detected left adrenal gland focal lesion previously qualified for surgical treatment was admitted to the Endocrinology Department in January 2024 for preoperative hormonal evaluation. In the patient’s history, an unintentional weight loss (approximately 15 kg over several months) and non-specific abdominal pain for a few weeks were present. An ultrasound examination performed in October 2023 for the listed symptoms visualized the aforementioned lesion of mixed echogenicity in the left adrenal gland, with dimensions of 90 × 61 mm. Outpatient abdominal MRI performed in December 2023 revealed a polycyclic nodular lesion of the left adrenal gland, measuring 85 × 83 × 83 mm, with intermediate signal in T1 and T2 images, heterogeneously enhancing after administration of a contrast agent, with areas of central necrosis—an image suggestive of ACC/pheochromocytoma. The patient has not systematically monitored her blood pressure at home. However, in the results presented by the patient from several days prior to hospitalization, systolic blood pressure (BP) values exceeded 160 mmHg, which allowed the diagnosis of hypertension. Due to suspected secondary etiology of hypertension, treatment with doxazosin was implemented. The patient denied other symptoms suggestive of a pheochromocytoma. On physical examination, she did not present typical clinical features of overt Cushing’s syndrome (CS) and other endocrine stigmatization hallmarks.

**Figure 1:**
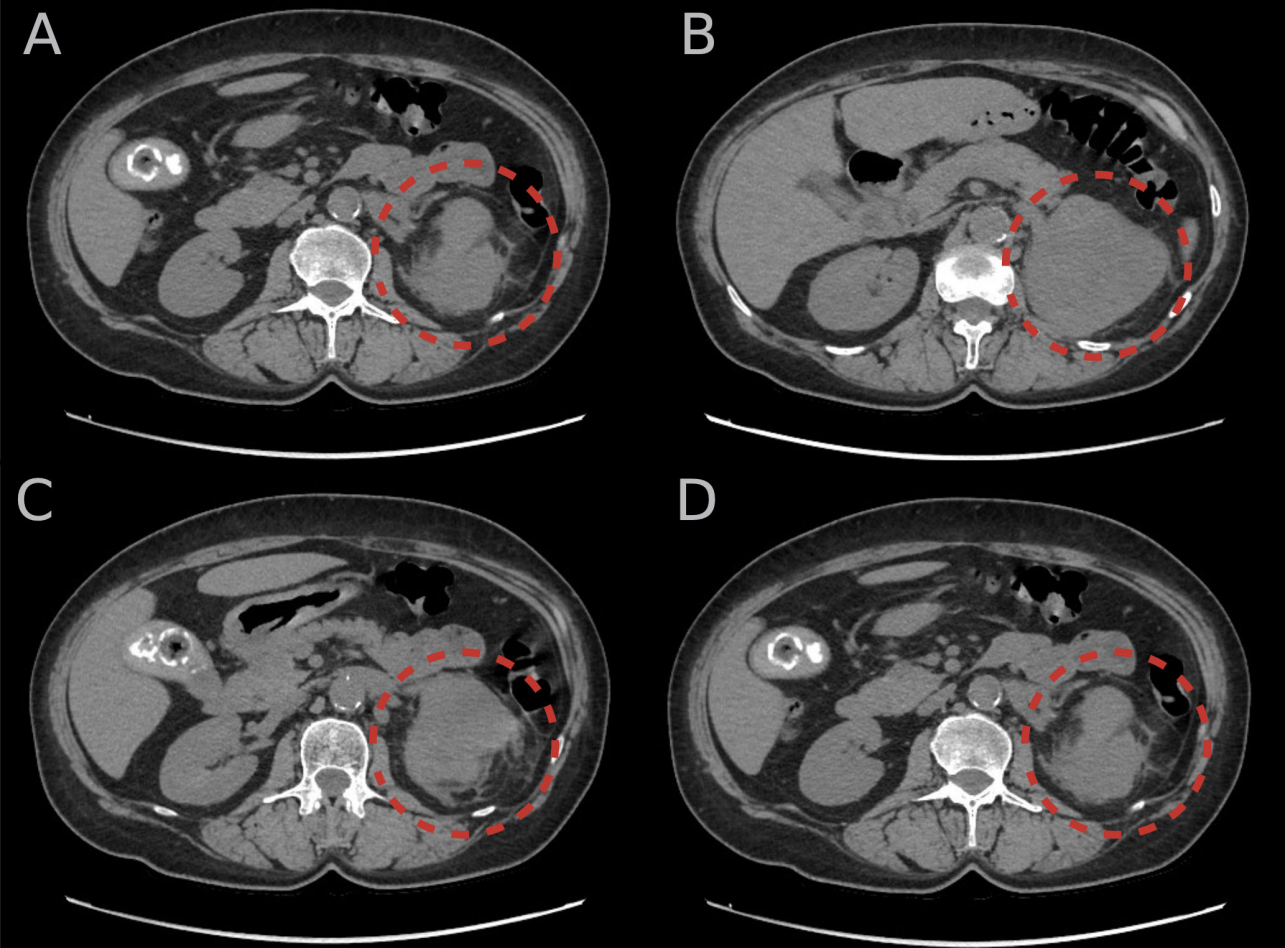
**(A–D)** An unenhanced computer tomography scans of the abdomen showing solid, heterogeneous tumor in the left adrenal gland (red circular lines) measuring approximately 87 × 64 × 107 mm with a density of approximately 35 Hounsfield units.

Throughout the hospital stay, the hormonal evaluation confirmed a mild autonomic cortisol secretion (MACS)—morning serum cortisol: 20.5 (URL 19.4) μg/dL, midnight serum cortisol: 6.9 (URL 5.4) μg/dL, adrenocorticotropin: 12.95 (URL 48) pg/mL, serum cortisol after 1 mg of dexamethasone: 4 (URL 1.8) μg/dL, dehydroepiandosterone sulfate: 77.1 (URL 182.2) μg/dL. In twice 24-h urine collection, a significantly elevated (4.5–6 × URL) excretion of 3MT with normal concentrations of metanephrine and normetanephrine was found. Other laboratory assays did not indicate any significant abnormalities: 17-hydroxyprogesterone: 0.87 (URL 0.9) ng/mL and total testosterone 0.72 (URL 1.24) nmol/L. The potential influence of non- and pharmacological factors on the metoxycatecholamine measurement was excluded. An unenhanced CT scan was performed to control the dimensions of the lesion and visualized a solid, heterogeneous tumor in the left adrenal gland field measuring about 87 × 64 × 107 mm with a density of approximately 35 HU (**Figure 1**).

The patient was referred for ^18^F-FDG-PET-CT, which revealed a metabolically active nodular lesion measuring 93 × 79 × 94 mm with a maximal SUV of 27.3 originating from the left adrenal gland in the retroperitoneal cavity. No metabolic active metastatic lesion was found in this examination. Due to suboptimal BP control, the dose of doxazosin was escalated (4 mg, taken two times a day). At the end of January 2024, after the required pharmacological preparation, a total left adrenalectomy with simultaneous splenectomy and regional lymphadenectomy was performed. A postoperative hormonal evaluation did not show adrenal insufficiency. Clinically, after surgery, the patient required continued hypotensive treatment at a reduced doses (doxazosin, 1 mg daily).

Histopathological evaluation identified adrenocortical carcinoma pT3N0. The preparation exhibited a high-grade mitotic tumor index and a 7/7 score on the modified Weiss scale. The microscopic assessment revealed high-grade nuclear atypia of epithelioid and pleomorphic cells, forming a solid tumor, with necrosis and small hemorrhages. Tumor invasion was also found in small vessels surrounding and across the organ capsule. Immunohistochemical (IHC) staining was negative for inhibin, calretinin, chromogranin, synaptophysin, and neuron-specific enolase (NSE). The Ki-67 index was approximately 80%. No cancer metastasis was found in the sampled lymph nodes. A subsequent histopathological verification, also performed by another experienced pathomorphologist, resulted in an 8/9 Weiss score and a 7/7 score according to Aubert’s modified Weiss system. IHC evaluation yielded a positive reaction for CKPAN, Vimentin, CD10, GATA-3, and S100 (in part of the cells). Markers of adrenal cortical differentiation, or markers typical of the conventional form of ACC, were negative; however, single, diffuse atypical cells exhibited a positive reaction for calretinin, which may indicate cortical differentiation of some of the tumor cells. Although not entirely characteristic and typical, the morphological picture and immunophenotype may correspond to the “sarcomatoid” subtype of adrenocortical carcinoma. The decision about adjuvant therapy with mitotane was made by the oncology team, and the patient has undergone this treatment.

## Discussion

The most common clinical manifestations of ACC include symptoms resulting from excess hormones, mainly cortisol, hence plethora, muscle atrophy, or metabolic complications such as diabetes mellitus (DM) or osteoporosis (4). The next most frequently produced by ACC hormones are adrenal androgens, causing hirsutism, and virilization (4). Hyperaldosteronism remains the fewest endocrine disorder in ACC, and the hypokalemia that may occur in this group of patients is more likely to arise from severe hypercortisolemia, as excess of cortisol affects as many as 50%–70% of ACC patients (2). It is worth mentioning the possibility of combined endocrine disorders in patients with adrenal tumors, which clinically may be reflected in an increased possibility of ACC (2). However, the presented patient did not exhibit clinical signs of overt CS but rather an abdominal discomfort that may result from the dimensions of the adrenal lesion—the tumor to one extent measured above 10 cm. ACCs are usually large tumors at the time of diagnosis, usually exceeding 6 cm in diameter (4). Thus, the clinical picture of an ACC patient may also include symptoms secondary to the effect of the tumor mass, mainly mentioned abdominal pain, as well as a feeling of abdominal fullness or early satiety (4). Symptoms resulting from the tumor mass effect usually emerge in hormonally inactive ACC cases (2). Noteworthily, ACC usually occurs unilaterally, while bilateral lesions, especially in patients with active malignancy, may suggest metastatic adrenal lesions (2).

In the presented patient, MACS was diagnosed. Although MACS patients are not considered to be at high risk of developing overt CS, it is recommended to actively search for potential cortisol-related comorbidities, such as hypertension or type 2 DM (1). In our patient, glycated hemoglobin was 5.7%, and further observation was planned.

Most patients with pheochromocytoma present with paroxysmal symptoms such as palpitation, sweating, anxiety attacks, or hypertension that poorly responds to conventional treatment, even leading to hypertensive crises (7). Although most pheochromocytoma tumors secrete excess norepinephrine, it is essential to mention the rare epinephrine-secreting tumors, which are associated with hypotension and shock after alpha-blocker administration caused by the solid beta-adrenergic effects of epinephrine (7). Alpha-blockers are medications preventing hypertensive crises during surgical treatment (7). As mentioned in the presentation, the patient was diagnosed with hypertension, and due to the clinical suspicion of pheochromocytoma, pharmacotherapy with doxazosin was implemented. After adrenalectomy, hypertension persisted in the presented patient, which supports that it did not result from excess secretion of the catecholamines by the tumor. However, there are some doubts: in our patient, of the three metoxycatecholamine metabolites tested in twice 24-h urine collection, only 3MT excretion was significantly elevated (4.5–6 × URL). The 3MT is a metabolite of dopamine metabolism, which is often excreted by extra-adrenal lesions (8). Postoperative evaluation of our patient has shown a normalization of 3MT.

In a similar case to ours, described by Ni and Htet, the 28-year-old female patient also did not present symptoms suggestive of CS or virilization but reported those indicative of pheochromocytoma, namely, palpitations, excessive sweating, flushing, and cold extremities, which correlated with high vanillylmandelic acid (VMA) urine excretion (3 × URL) which had been normalized after adrenalectomy, but the histopathological examination of resected tumor showed an ACC (9).

In the absence of clinical features of steroid hormone production, it is advisable to biochemically exclude pheochromocytoma (4) by measuring plasma-free metanephrines or fractionated urinary metanephrines in all patients with adrenal lesions not presenting typical adenomas features (1). It has been indicated that in patients without clinical signs suggestive of pheochromocytoma, determining plasma fractions provides higher sensitivity than assessing urinary metanephrines. Despite considering the determination of plasma-free metanephrines as the most sensitive currently available diagnostic assay, the rate of false-negative results is estimated at 2.1%. False-positive results can be induced by either clinical conditions (heart failure, anxiety, hypoglycemia, pain, sleep apnea syndrome, renal failure), medications (sympathomimetics, tricyclic antidepressants, atypical neuroleptics), stimulants (coffee, nicotine), or dietary factors (fruits, especially bananas) (10).

Alert signs, such as night sweats or cachexia, and paraneoplastic syndromes in the course of ACC are rather rare (4). However, cases of cancer-related hypoglycemia in patients with ACC have been described (11) or hyperrenin hyperaldosteronism (12) and erythropoietin-related polycythemia (13). The presented patient, however, reported significant unintentional weight loss.

The pathomorphological diagnosis of ACC remains a diagnostic challenge due to its rarity (14) and the presence of several variants—conventional, pediatric, oncocytic, myxoid, and sarcomatoid (15, 16). Biopsy plays a limited role in the diagnosis of adrenal tumors, being contraindicated in the majority of cases because of the risk of tumor dissemination and the possibility of inducing a catecholamine crisis in the case of pheochromocytoma. However, it acquires significance in adrenal lesions with a possible metastatic manifestation of extra-adrenal tumors (3). Evaluation of Ki-67, one of the most relevant factors of proliferation, remains crucial, while its assessment in biopsy material may not reflect the index titer in the entire tumor (2). For the proper pathomorphological evaluation, several tools may be used: the Weiss system, the reticulin algorithm, the Helsinki system, and the Lin–Weiss–Bisceglia system (2, 17). In general clinical practice, the diagnosis of malignant neoplasm is based on finding at least three of nine morphological parameters on light microscopy, according to the Weiss scoring system (3, 15). In the case of our patient, histopathological verification was firmly supportive of ACC, i.e., high-grade mitotic tumor index, nuclear atypia, presence of necrosis and hemorrhages, and vascular invasion. Specific ACC markers (inhibin and calretinin) were negative in the first IHC determination, and positive, only for calretinin in some of the diffuse atypical cells in the reevaluation, so the sarcomatoid subtype of ACC was diagnosed. This subtype is the least common variant of ACC, characterized by aggressive behavior and a worse prognosis than other ACC variants (18). In a study by Papathomas et al. showing six cases of sarcomatoid ACC, obtained from five pathomorphology departments, SF-1 was the only adrenal cortex marker positive in the epithelial component in all cases, but was always negative in the sarcomatoid areas; cadherins were positive only in the epithelial component (18). In our patient’s case, SF-1 and cadherin were not determined, but as reported above, they may be negative in the sarcomatoid components of the tumors. The histologic and immunohistochemical findings also ruled out the possibility of pheochromocytoma. Conversely, the indicated ambiguous immunohistochemistry results do not oppose the diagnosis of ACC and may be helpful in determining the prognosis. In the study conducted by Zlatibor et al. involving 30 ACC cases, among subjects with negative inhibin staining, half of them died within the first 6 months, and only one person in this group survived longer than 1 year (19). In that study, a similar relationship between survival length and a worse prognosis was also observed with a Ki-67 index ≥7% (19).

Collected data highlight the predominance of diagnoses of advanced ACC cases with distant metastases (stage 4) in the past; now, it is reported that 25% to 30% of patients with ACC present with metastases (4). The most common locations for metastatic lesions include the lungs, liver, and bones (4). A complete staging evaluation, including at least a chest CT and ^18^F-FDG-PET-CT, is recommended before surgical treatment (1). In our patient, the staging was performed, and no metastases were found.

Reviewing the literature, we found a few similar cases besides those mentioned above, some of which have been dated distantly and thus may not contain all the clinical data. A summary of the described cases is presented in **Table 1**; however, several of them are worth additional emphasize. In the paper by Alsabeh et al., five patients with elevated catecholamine secretion in urine or serum were described and the histopathological evaluation confirmed two cases of ACCs and three cases of adrenal cortical adenomas (ACAs) (20). Both cases with a diagnosis of ACC involved men with previously diagnosed hypertension. In each, distant metastases were found in the months following adrenalectomy (20). In both ACC cases, as well as in one ACA case, immunohistochemical evaluation revealed positive staining for NSE and synaptophysin and negative for chromogranin (20). Another case worth emphasizing is the patient described by Fassina et al. in which, due to further diagnostics because of ongoing left-sided pain symptoms, slides obtained from a performed 7-month-earlier laparotomic excision of a pheochromocytoma in the left adrenal gland were reevaluated, which resulted in a final diagnosis of coexisting pheochromocytoma and ACC (21). The patient was presumed to be genetically predisposed to develop endocrine dysplasia, but germline mutations in VHL, RET, SDHB, SDHC, and TMEM127 were excluded, implying sporadic co-occurrence of the tumors in mentioned case (21). Since pheochromocytoma may produce adrenocorticotropin, insulin-like growth factor 2, somatostatin, growth hormone-releasing hormone, corticotropin-releasing hormone, and interleukin 6, it was considered, however, that it might also stimulate the adrenal cortex to hyperfunction and proliferation through the paracrine pathway (21).

**Table 1:** Table presenting an overview of published clinical cases of adrenocortical carcinoma manifesting as pheochromocytoma (9, 20–28).

Case authors	Patient age/gender	Symptoms	Imaging findings	Laboratory evaluation	Histopathology/immunohistochemistry assessment	Additional information
Walters et al. (22).	44/M	History of exertional dyspnea for 3 months and one severe attack of nocturnal dyspnea Paroxysmal hypertension	Intravenous pyelography: large mass in the upper pole of the left kidney	24-h urine collection was positive for catecholamines, VMA, and 17(OH)CS Hypercortisolism: not mentioned Hyperaldosteronism: not mentioned Hypokalemic alkalosis	The tumor weighed 840 g, was encapsulated, and consisted of necrotic material and hemorrhagic areas. The capsule was unaffected. The tumor was removed incompletely.	A malignant tumor of the left adrenal was present, and during surgery BP increased and required continuous phentolamine infusion. After removal of the tumor, NE was required for several days to maintain proper BP. Catecholamines were extracted from the tumor (200 mg/g tissue), although histologically it had the appearance of a cortical tumor, and a later autopsy demonstrated atrophy of the contralateral adrenal cortex.
Alsabeh et al. (20)	67/M	History of hypertension for 10–15 years	CT: 40 mm left adrenal heterogeneous mass with central necrosis ^123^I-MIBG scintigraphy: no evidence of extra-adrenal pheochromocytoma	D: 56 pg/mL (NRV: 0–10) E: 190 pmol/L (NRV: 54 pmol/L–1.091 pmol/L) NE: 6.8 nmol/L (NRV:0.52–3.07) Hypercortisolism: not mentioned Hyperaldosteronism: not mentioned 15 months after left adrenalectomy: increased 24-h urinary 17-ketosteroids, MN, and NMN (values not mentioned); normal concentrations of urinary catecholamines	25 mm ACC	Digital imaging performed during workup for prostatic adenocarcinoma
Robinson and Baker-Bates (23)	29/M	Recurrent pain on the left side of the chest and abdomen for 5 years 9-year history of vomiting, unspecified weight loss, and increased sweating Paroxysmal hypertension Variable fasting blood glucose	Retrograde pyelogram: slight rotation of the left kidney No other imaging studies available (a clinical case from a distant date)	No data available to assess catecholamine levels in this case	Extra-peritoneal exploration of the left kidney region: large tumor situated superiorly to the left kidney Macroscopically: partially encapsulated, oval tumor measuring 130 × 60 × 70 mm, with areas of necrosis and hemorrhage The chromaffin reaction was negative. Histopathology: ACC	The third episode of the listed symptoms was classified as pericarditis, and a tuberculous background was suspected because the patient belonged to the military. During the operation, BP varied between 120/90 mmHg and 260/170 mmHg.
Fassina et al. (21)	46/F	Pain in the left flank	Not mentioned	Not mentioned	Laparoscopic biopsy from the left inferior kidney pole: cytology findings suggestive of an adrenal cortical origin Re-evaluation of histological slides: coexisting pheochromocytoma and ACC Immunochemistry: Inhibin α—positive CD56—positive Chromogranin A—negative Synaptophysin—negative Serotonin—negative Somatostatin—negative CK7—negative CK20—negative	7 months earlier, the patient had undergone laparotomic excision of a pheochromocytoma in the left adrenal gland. Due to the recurrence of the symptoms, the diagnosis was expanded and the histological slides from that time were reevaluated.
Ni and Htet (9)	28/F	Palpitations Excessive sweating and flushing Cold extremities Dizziness History of unspecified weight loss in 1.5 years Hypertension	Ultrasound of abdomen: right supra-renal mass of mixed echogenicity measuring 131 mm × 77 mm Abdominal CT: a well-circumscribed soft tissue mass of 165 × 65 × 87 mm in the right retroperitoneal region with calcifications	Hypercortisolism (–) Hyperaldosteronism—not mentioned Urinary VMA: 21 mg/24 h (NRV 1.8–7.1 mg/24 h)	Histopathology: encapsulated adrenal tumor arising from the cortex, with areas of hemorrhage, calcification, and necrosis. A moderate degree of atypia was noted, consisting of giant cells and spindle cells. No invasion into the adrenal vein or lymphatics. The impression of biopsy result was ACC. Immunohistochemistry: staining cannot be performed (limited resources).	Physical examination revealed a palpable lesion in the right lumbar region measuring approximately 50 × 50 mm, firm in consistency and ballotable. Re-check urinary VMA two weeks after surgery was 5.4 mg/24 h (NRV 1.8 mg–7.1 mg/24 h).
Jain et al. (24)	45/F	Complaints of vague abdominal pain for 3 years Headache Episodes of palpitations, sweating and vertigo Hypertension diagnosis	Abdominal CE-CT: a right suprarenal tumor measuring 60 × 50 × 50 mm with solid cystic components and fluid levels suggestive of intratumoral hemorrhage Abdominal MRI: multiple cystic spaces of various sizes in the area of the right adrenal gland suggestive of pheochromocytoma	Serum cortisol and 24-h urinary cortisol were normal 24-h VMA 8.8 mg (NRV: 1.8 mg–7 mg/24 h) Other tests could not be undertaken (limited resources)	Histopathology: ACC	Per abdomen, a 50 × 50 mm ballotable solid—cystic mass overlying the right kidney
Nowak et al. (25)	39/M	Uncontrolled hypertension	NC-CT: 63-mm left adrenal tumor, 35 HU CE-CT: APW 60%, RPW 30%—left adrenal tumor Abdominal MRI: no signal loss in the out-of phase ^123^I-MIBG scintigraphy: increased heterogeneous uptake in the left adrenal gland SRS: increased heterogeneous uptake in the left adrenal gland	Hypercortisolism (–): Hyperaldosteronism (–): Urinary metoxycatecholamines: 916 μg/24 h and 953 μg/24 h (URL 1,000 μg/24 h)	Macroscopically: 71-mm tumor Histopathology: ACC Immunohistochemistry: chromogranin-negative type 2 and type 5 somatostatin receptors - positive	Simultaneously, right adrenal lesion 23 mm in diameter, APW 98% and 77% RPW was also imaged in C-CT
Shetty et al. (26)	57/F	At the age of 47 patient developed symptoms of flushing and hypertension;	Abdominal MRI (Feb 2013): 14 × 9 mm left adrenal mass that lacked T2 hyperintensity CE-CT (Jul 2013): left adrenal mass measuring 23 × 29 × 19 mm with 91.7 HU on post-contrast imaging Abdominal MRI, CT of the chest/abdomen/pelvis (Sep 2015): multiple lung nodules	First biochemical evaluation: normal concentrations of catecholamines, MN, free cortisol, and VMA.	First histopathology: ACC/pheochromocytoma Second histopathology: ACC Immunohistochemistry: vimentin—positive synaptophysin—positive calretinin—positive chromogranin—negative desmin—negative inhibin—negative CD 34, CK7, CAM5.2, D2-40, GATA 3—negative SF-1—positive TTF1—negative Ki-67: 70%–80%	Patient with Lynch Syndrome The patient subsequently underwent a left adrenalectomy, and pathology results showed pheochromocytoma with a PASS of 11 out of 20. She had resolution of her symptoms following surgery and underwent surveillance scans every 3–6 months. However, she had recurrence of symptoms in 2014 reported as asthenia and migraine headaches, but without radiological features of recurrence.
Ali and Mirza (27)	65/F	History of osteoporotic hip fracture Few years later abdominal pain and fever	Abdominal CT: 30 × 24 mm left adrenal mass—37 HU pre-contrast, 125 HU post-contrast, 74 HU during the delayed contrast phase Abdominal CT after few years: left adrenal lesion dimensions had increased to 96 × 77 mm	24-h urine collection positive for MN, E, and NE	Surgical pathology revealed an ACC Low-grade, without lymphatic or vascular invasion the tumor was confined to the adrenal cortex without invasion through the tumor capsule	Patient disappeared from follow-up for many years, reappeared due to described symptoms.
Kiliani et al. (28)	52/F	Flushing Panic attack Psychosis Episodes of acute anxiety, hallucinations Palpitations Paroxysmal hypertension Tachycardia A 9-kg weight loss over the 3 - 4 months	CT of the abdomen and pelvis: right adrenal heterogeneous mass with calcifications Abdominal CT performed with adrenal protocol: 45 × 37 × 46 mm mass in the right adrenal gland, with density 38 HU on precontrast imaging, 68 HU at 90-s delay, and 98 HU at 10-min delay. The mass contained several calcifications, suggesting degeneration or necrosis.	Negative serum and urinary MN and NMN Hyperaldosteronism (–): Elevated 24-h UFC, elevated serum AM cortisol, and positive dexamethasone suppression test.	Histopathology: ACC	After surgery, psychiatric symptoms resolved.

The articles summarized in the table have been placed in order of publication. M, male; F, female; VMA, vanillylmandelic acid; 17(OH)CS, 17(OH) corticosteroids; BP, blood pressure; CT, computer tomography; ^123^I-MIBG, ^123^I-metaiodobenzylguanidine; NRV, normal reference values; D, dopamine; E, epinephrine; NE, norepinephrine; MN, metanephrines; NMN, normetanephrines; ACC, adrenal cortex carcinoma; CE-CT, contrast-enhanced computer tomography; MRI, magnetic resonance imaging; HU, Hounsfield units; APW, absolute percentage washout; RPW, relative percentage washout; SRS, somatostatin-receptor scintigraphy; URL, upper reference limit; NC-CT, non-contrast computer tomography; PASS, Pheochromocytoma of the Adrenal gland Scaled Score; UFC, urinary free cortisol.

## Conclusions

As we have summarized in our paper, ACC is among the rare neoplasms with challenging pathomorphological evaluation and multiple clinical manifestations, including those mimicking pheochromocytoma. Although our review may have some limitations, such as some clinical cases dating to distant years (the 1960s), when the possibilities of radiological and hormonal evaluation were limited, most provide solid clinical data supporting such rare ACC presentation. The necessity of including these distant in time cases in our review only confirms how infrequent the reported manifestation of ACC is and how extensive a literature search may be confronted by clinicians struggling with comparable concerns while diagnosing patients with adrenal tumors of equivocal clinical presentation. Based on the ambiguities in our case report and the available papers referenced from the literature, we recommend managing this group of patients with a comprehensive multidisciplinary team of clinicians using the available laboratory and imaging modalities. The case presented here emphasizes the importance of an individualized approach in caring for patients with adrenal tumors. Overall, the case we describe is one of the few in which we have obtained laboratory, imaging, histopathologic, and immunohistochemical results, which provides a further basis for advancing our knowledge of this rare presentation of ACC.

## References

[1] Fassnacht M Tsagarakis S Terzolo M Tabarin A Sahdev A Newell-Price J. European Society of Endocrinology clinical practice guidelines on the management of adrenal incidentalomas, in collaboration with the European Network for the Study of Adrenal Tumors. Eur J Endocrinol. (2023) 189:G1–42. doi: 10.1093/ejendo/lvad066 37318239

[2] Handkiewicz-Junak D Dedecjus M Ambroziak U Barczyński M Bednarek-Papierska L Chmielik E. Polish diagnostic and therapeutic recommendations for adrenocortical carcinoma. Endokrynol Polska. (2024) 75:339–58. doi: 10.5603/ep.101677 39279304

[3] Fassnacht M Assie G Baudin E Eisenhofer G de la Fouchardiere C Haak HR. Adrenocortical carcinomas and Malignant phaeochromocytomas: ESMO-EURACAN Clinical Practice Guidelines for diagnosis, treatment and follow-up. Ann Oncol. (2020) 31:1476–90. doi: 10.1016/j.annonc.2020.08.2099 32861807

[4] Else T Kim AC Sabolch A Raymond VM Kandathil A Caoili EM. Adrenocortical carcinoma. Endocr Rev. (2014) 35:282–326. doi: 10.1210/er.2013-1029 24423978 PMC3963263

[5] Kamilaris CDC Hannah-Shmouni F Stratakis CA. Adrenocortical tumorigenesis: Lessons from genetics. Best Pract Res Clin Endocrinol Metab. (2020) 34:101428. doi: 10.1016/j.beem.2020.101428 32507359 PMC7427505

[6] Ahuja K Goudar R. A novel lynch syndrome kindred with hereditary adrenal cortical carcinoma. Cancer Genet. (2024) 288–289:137–40. doi: 10.1016/j.cancergen.2024.11.005 39571462

[7] Walther MM Keiser HR Linehan WM. Pheochromocytoma: evaluation, diagnosis, and treatment. World J Urol. (1999) 17:35–9. doi: 10.1007/s003450050102 10096149

[8] Eisenhofer G Goldstein DS Sullivan P Csako G Brouwers FM Lai EW. Biochemical and clinical manifestations of dopamine-producing paragangliomas: utility of plasma methoxytyramine. J Clin Endocrinol Metab. (2005) 90:2068–75. doi: 10.1210/jc.2004-2025 15644397

[9] Ni H Htet A. Adrenal cortical carcinoma masquerading as pheochromocytoma: a case report. Ecancermedicalscience. (2012) 6:277. doi: 10.3332/ecancer.2012.277 23152728 PMC3493057

[10] Eisenhofer G Pamporaki C Lenders JWM. Biochemical assessment of pheochromocytoma and paraganglioma. Endocr Rev. (2023) 44:862–909. doi: 10.1210/endrev/bnad011 36996131

[11] Ishikura K Takamura T Takeshita Y Nakagawa A Imaizumi N Misu H. Cushing’s syndrome and big IGF-II associated hypoglycaemia in a patient with adrenocortical carcinoma. BMJ Case Rep. (2010) 2010:bcr07.2009.2100. doi: 10.1136/bcr.07.2009.2100 PMC3027795 22461853

[12] Yamanaka K Iitaka M Inaba M Morita T Sasano H Katayama S. A case of renin-producing adrenocortical cancer. Endocr J. (2000) 47:119–25. doi: 10.1507/endocrj.47.119 10943735

[13] Oka T Onoe K Nishimura K Tsujimura A Sugao H Takaha M. Erythropoietin-producing adrenocortical carcinoma. Urol Int. (1996) 56:246–9. doi: 10.1159/000282852 8776824

[14] Aiba M Fujibayashi M. Histopathological diagnosis and prognostic factors in adrenocortical carcinoma. Endocr Pathol. (2005) 16:13–22. doi: 10.1385/EP:16:1:013 16000842

[15] Papotti M Libè R Duregon E Volante M Bertherat J Tissier F. The Weiss score and beyond–histopathology for adrenocortical carcinoma. Horm Cancer. (2011) 2:333–40. doi: 10.1007/s12672-011-0088-0 PMC10358021 21997290

[16] Mete O Erickson LA Juhlin CC de Krijger RR Sasano H Volante M. Overview of the 2022 WHO classification of adrenal cortical tumors. Endocr Pathol. (2022) 33:155–96. doi: 10.1007/s12022-022-09710-8 PMC8920443 35288842

[17] Gambella A Volante M Papotti M. Histopathologic features of adrenal cortical carcinoma. Adv Anat Pathol. (2023) 30:34–46. doi: 10.1097/PAP.0000000000000363 36084635

[18] Papathomas TG Duregon E Korpershoek E Restuccia DF van Marion R Cappellesso R. Sarcomatoid adrenocortical carcinoma: a comprehensive pathological, immunohistochemical, and targeted next-generation sequencing analysis. Hum Pathol. (2016) 58:113–22. doi: 10.1016/j.humpath.2016.08.006 27589897

[19] Zlatibor L Paunovic I Zivaljevic V Dundjerovic D Tatic S Djukic V. Prognostic significance of immunohistochemical markers in adrenocortical carcinoma. Acta Chir Belg. (2020) 120:23–9. doi: 10.1080/00015458.2018.1543822 30499377

[20] Alsabeh R Mazoujian G Goates J Medeiros LJ Weiss LM. Adrenal cortical tumors clinically mimicking pheochromocytoma. Am J Clin Pathol. (1995) 104:382–90. doi: 10.1093/ajcp/104.4.382 7572786

[21] Fassina A Cappellesso R Schiavi F Fassan M. Concurrent pheochromocytoma and cortical carcinoma of the adrenal gland. J Surg Oncol. (2011) 103:103–4. doi: 10.1002/jso.v103.1 21031430

[22] Walters G Wyatt GB Kelleher J. Carcinoma of the adrenal cortex presenting as a pheochromocytoma: report of a case. J Clin Endocrinol Metab. (1962) 22:575–80. doi: 10.1210/jcem-22-6-575 14004782

[23] Robinson PL Baker-Bates ET. Adrenal cortical carcinoma simulating a phæochromocytoma. Br J Surgery. (2005) 41:399–403. doi: 10.1002/bjs.18004116818 13126484

[24] Jain S Agarwal L Nadkarni S Ameta A Goyal A Kumar R. Adrenocortical carcinoma posing as a pheochromocytoma: a diagnostic dilemma. J Surg Case Rep. (2014) 2014:rju030. doi: 10.1093/jscr/rju030 24876502 PMC4017231

[25] Nowak KM Łebek-Szatańska A Samsel R Roszkowska-Purska K Ćwikła JB Papierska L. Adrenocortical carcinoma mimicking pheochromocytoma on iodine 123-labeled metaiodobenzylguanidine scintigraphy. Pol Arch Intern Med. (2019) 129:822–3. doi: 10.20452/pamw.14958 31469118

[26] Shetty I Fuller S Raygada M Merino MJ Thomas BJ Widemann BC. Adrenocortical carcinoma masquerading as pheochromocytoma: a histopathologic dilemma. Endocrinol Diabetes Metab Case Rep. (2020) 2020:19–0147, HYPERLINK "https://pubmed.ncbi.nlm.nih.gov/?term=%22Shetty%20I%22%5BAuthor%5D" Impana Shetty 1, HYPERLINK "https://pubmed.ncbi.nlm.nih.gov/?term=%22Fuller%20S%22%5BAuthor%5D" Sarah Fuller 1, HYPERLINK "https://pubmed.ncbi.nlm.nih.gov/?term=%22Raygada%20M%22%5BAuthor%5D" Margarita Raygada 1, HYPERLINK "https://pubmed.ncbi.nlm.nih.gov/?term=%22Merino%20MJ%22%5BAuthor%5D" Maria J Merino 2, HYPERLINK "https://pubmed.ncbi.nlm.nih.gov/?term=%22Thomas%20BJ%22%5BAuthor%5D" B J Thomas 1, HYPERLINK "https://pubmed.ncbi.nlm.nih.gov/?term=%22Widemann%20BC%22%5BAuthor%5D" Brigitte C Widemann . doi: 10.1530/EDM-19-0147 PMC6993251 31917677

[27] Ali M Mirza L. An unusual case of Adrenocortical Adenocarcinoma with Biochemical Masquerade of Pheochromocytoma. Pak J Med Sci. (2021) 37:1241–3. doi: 10.12669/pjms.37.4.3916 PMC8281192 34290815

[28] Kilani Y Mon AM Laxamana T Kamal SAF Zain R Sohail H. An unusual presentation of adrenocortical carcinoma (ACC): panic attacks and psychosis. Am J Case Rep. (2022) 23:e937298. doi: 10.12659/AJCR.937298 36037151 PMC9438937

